# Social capital and public health: responding to the COVID-19 pandemic

**DOI:** 10.1186/s12992-020-00615-x

**Published:** 2020-09-25

**Authors:** Anna S. Y. Wong, Jillian C. Kohler

**Affiliations:** 1 World Health Organization Collaborating Centre for Governance, Accountability, and Transparency in the Pharmaceutical Sector, 144 College St, Toronto, Ontario M5S 3M2 Canada; 2 University of Toronto Leslie Dan Faculty of Pharmacy, 144 College St, Toronto, Ontario M5S 3M2 Canada

**Keywords:** Social capital, Health policy, COVID-19, Mental health, Health access

## Abstract

**Background:**

As countries continue to respond to the COVID-19 pandemic, the importance of ensuring that fair and equal access to healthcare for all is more urgent than ever. Policies that promote social capital building along all levels of society may offer an important avenue for improved healthcare delivery and health systems strengthening in the COVID-19 response.

**Main body:**

In reference to the established and emerging literature on social capital and health, we explore the role of social capital in the COVID-19 health policy response. We analyse current research with respect to mental health, public health policy compliance, and the provision of care for vulnerable populations, and highlight how considerations of bonding, bridging, and linking capital can contribute to health systems strengthening in the context of the COVID-19 response and recovery effort.

**Conclusions:**

This article argues that considerations of social capital – including virtual community building, fostering solidarity between high-risk and low-risk groups, and trust building between decision-makers, healthcare workers, and the public – offer a powerful frame of reference for understanding how response and recovery programs can be best implemented to effectively ensure the inclusive provision of COVID-19 health services.

## Background

The global response to COVID-19 has required decisiveness, resilience, and resolve from governments around the world. However, economic, legal, technological, geographic, and cultural barriers can limit the ability of a government to effectively respond to critical public health needs. The intricate network of stakeholders that operate within and interconnect with the public health space is an essential component of a health system’s response. In this context, considerations of social capital emerge as a powerful frame of reference for understanding how health interventions may be best implemented to effectively ensure an inclusive extension of health services for all members of society. It is patently clear that if a population group is excluded from accessing the health system and its attendant services and products, the efficacy of any pandemic response or recovery program may be severely undermined.

Putnam conceptualizes social capital as *the behaviour of social networks and relationships, characterized by the qualitative presence of enhanced trust and reciprocity* [1]. Within the field of public health, social capital has been studied by many scholars as both a variable to improve health outcomes and as a framework for assessing public health interventions [2]. Bonding, bridging, and linking capital describe three sub-types of social capital that are particularly prescient in the context of public health studies. Bonding capital describes the social capital derived from the social networks and relationships within homogenous groups, bridging capital from those within heterogeneous groups comprised of members of equal power or authority (‘horizontal’ capital), and linking capital from those within heterogeneous groups comprised of members ordered along an explicit, formal, or institutionalized gradient of power or authority (‘vertical’ capital) [1]. Expressed through the creation of cultures of obligation or expected reciprocity, enhanced community-based information channels, or the establishment of informal codes of socially normative behaviour, social capital may benefit members of a community by encouraging solidarity, expediting knowledge dissemination, and facilitating the social integration of previously excluded members [3].

## Social capital and COVID-19: challenges and opportunities

As countries adopt urgent public health measures in response to the many challenges posed by COVID-19, lessons learned from public health intervention studies that link enhanced social capital with improved mental health outcomes, greater community buy-in, and the extension of health services to vulnerable populations suggest a critical role for social capital in ensuring a rapid adjustment to today’s new public health reality.

Preliminary studies conducted in the United States lend support to the importance of social capital in the COVID-19 response, with the growth rate of new COVID-19 cases found to be negatively associated with the amount of social capital at both the state and county levels [4]. However, explicitly seeking to employ or strengthen existing social networks for the purpose of improved health outcomes demands thoughtful consideration as to the nature of each intervention group’s social layout. Distinct structures of social interaction may behave differently in response to a particular public health intervention, and thus, it is imperative that a social capital-based response maintain the capacity to not only recognise scenarios in which social capital building initiatives may effectively complement the public health agenda, but also accurately identify the subtypes of social capital already present within communities. In this way, a social capital-centric public health approach to the COVID-19 response should not be understood as a one-size-fits-all policy supplement, but rather, as a framework for understanding how the social dynamics between discrete groups of actors can be employed to most efficiently implement pandemic-related health policies.

### Bonding capital

In countries around the world, social distancing policies have emerged as a central component of the COVID-19 response. Measures including post-exposure quarantining, shelter-in-place orders, and limits on the size of social bubbles have been credited with ‘flattening the curve’ and reducing the growth of new infection cases. However, these have also contributed to the dramatic disruption of normal patterns of social interaction, raising serious mental health concerns amongst the general population and high-risk groups alike. As social distancing measures continue to limit access to customary channels of social support – such as spending in-person time with family members, friends, and colleagues of different households – studies within communities affected by COVID-19 report consistent observations of elevated mental health concerns. For example, heightened levels of anxiety and depression have been reported in China, greater stress and mental morbidity have been observed in Iran, higher levels of fear and panic behaviour have been noted in Japan, and increased health anxiety has been studied in Canada [5].

Addressing the potential mental health consequences of social distancing measures thus necessitates a conscious inclusion of psychological interventions in the international pandemic response. This is particularly true for high-risk groups, such as the elderly, those with underlying health conditions, and front-line health workers, since these groups represent the populations that are most likely to adhere to stricter forms of social distancing [6]. In this respect, robust bonding capital between physically distant family and community members may help insulate high-risk individuals from social isolation by preserving their pre-existing social networks. Efforts that consciously seek to promote virtual or physically distant community building thus stand as immediately available options to mitigate against potential adverse mental health responses to social distancing-induced isolation. Similarly, with social distancing increasingly being treated as a long-term policy contingent on the development and delivery of a successful COVID-19 vaccine, it is imperative that the public health response recognise the increased likelihood of social dislocation among individuals and communities with weak bonding capital, and thus, their enhanced risk of experiencing adverse mental health outcomes.

### Bridging capital

Effectively containing the spread of COVID-19 demands a unified response from all members of society. This is particularly true with respect to preventative public health measures that intrinsically require broad public buy-in, such as physical distancing or public mask-wearing. However, with some groups of individuals perceived to be at significantly lower risk to the most severe symptoms of COVID-19, psychological tolerance to reduced levels of precaution may vary considerably within a given population. If expressed through the refusal to comply with public health directives, the efficacy of preventative public health measures may be severely undermined. In this way, perhaps one of the most challenging obstacles to achieving public health policy compliance in the context of COVID-19 lies in persuading individuals to make decisions according to the exposure tolerance of high-risk individuals – potentially to whom they have no personal connection – rather than according to their own personal willingness to incur risk. Indeed, while full public buy-in to public health directives is an undoubted prerequisite to an effective pandemic response, communities with weak bridging capital between high-risk and low-risk populations may find this difficult to achieve. Initiatives that aim to build social capital by fostering enhanced solidarity and empathy between high-risk and low-risk groups are thus particularly important to the COVID-19 response. A recent set of studies investigating the role of empathy on physical distancing and mask-wearing compliance reinforces the potential impact of such initiatives, finding that public motivation to adhere to these preventative public health measures is promoted when empathy for those most vulnerable to COVID-19 is elicited [7]. Extended to the future delivery of a COVID-19 vaccine, an emphasis on the collective remains imperative; policies that deliberately seek to build bridging capital between high-risk and low-risk groups may serve as an effective tool to combat vaccine hesitancy by enabling members of the general population to recognise that their personal immunization decisions intimately influence the safety of those in their communities who are unable to be vaccinated (ex. due to allergic intolerances).

### Linking capital

In comparison to authoritarian systems of government, where public adherence to strict pandemic response measures may be unilaterally compelled through the use or threat of excessive force, compliance with government-mandated health directives among democratic systems requires that the public embrace a set of pro-health social norms that can weather the spread of scientifically unsupported information and/or a lack of trust in national health leaders. Measures that protect linking capital between democratic public health authorities and members of the public thus cannot not be overlooked. To this end, the dissemination of transparent and accurate public health information, enforcing expectations of public health policy compliance among political leaders, policy consistency between domestic agencies and departments, and policy congruency with recognized international health organizations stand as actionable social capital-centric options that may enhance public faith in the legitimacy of national COVID-19 responses.

Equally important is the role of linking capital in ensuring that the needs of the vulnerable are not ignored. Particularly in the context of the current COVID-19 pandemic, it is vital that individuals can access lifesaving health services regardless of their socioeconomic or legal status, cultural identity, or geographic location. In Italy, the exclusion of irregular migrants from receiving free COVID-19 testing was shown to undercut the government’s pandemic response [8], illustrating the importance of an inclusive, equitable, and population-wide approach to the COVID-19 health response. In the United States, a fear of legal reprisal has been identified as a potential deterrent to care among undocumented immigrants [9], further underscoring the importance of actively seeking to include the ‘invisible’ members of society when formulating public health policy. A previous study of linking capital in Spain demonstrated how nurses with low financial resources could be used to increase trusting relationships between irregular migrants and healthcare providers to increase the overall accessibility of healthcare services [10]. With respect to the COVID-19 response, which demands resilience across all levels of the health system, this suggests the particularly powerful role of linking capital in extending the accessibility of both general and pandemic-related health services to members of vulnerable or marginalized communities.

## Conclusions

A social capital-centric public health response is one that recognises the limitations of top-down policy and the value of social networks in achieving desirable public health outcomes. As concerns over mental health, public health policy compliance, and equitable access to healthcare services and products continue to confront the global COVID-19 response, measures that integrate considerations of bonding, bridging, and linking capital stand as immediately available options for health systems strengthening. Efforts to address the ongoing pandemic increasingly demand the participation of actors across different nationalities, disciplines, socioeconomic backgrounds, and political identities. As such, we must acknowledge the inherent importance of social solidarity and trust in achieving today’s urgent public health goals.

## Data Availability

Data sharing is not applicable to this article as no datasets were generated or analysed during the current study.
